# DIAGNOSIS AND MANAGEMENT OF INTRADUCTAL PAPILLARY MUCINOUS NEOPLASMS: A NARRATIVE REVIEW BASED ON INTERNATIONAL GUIDELINES

**DOI:** 10.1590/S0004-2803.24612025-099

**Published:** 2026-07-20

**Authors:** Claudia Teresa CARVENTE, Henrique Carvente TOLEDO, Carlos Fischer de TOLEDO

**Affiliations:** 1 Universidade Federal de São Paulo - Escola Paulista de Medicina, Departamento de Medicina, Disciplina de Gastroenterologia, São Paulo, SP, Brasil.; 2 Universidade Federal de São Carlos, Departamento de Física, São Carlos, SP, Brasil

**Keywords:** Intraductal papillary mucinous neoplasms, pancreatic cysts, clinical guidelines, risk stratification, endoscopic ultrasound, magnetic resonance imaging, artificial intelligence, Neoplasias papilares mucinosas intraductais, cistos pancreáticos, diretrizes clínicas, estratificação de risco, ultrassonografia endoscópica, ressonância magnética, inteligência artificial

## Abstract

**Background:**

Intraductal papillary mucinous neoplasms (IPMNs) are the leading cause of incidentally detected pancreatic cysts and exhibit a wide biological spectrum with variable malignant potential. Recent advances in imaging, endoscopic assessment, molecular testing, and artificial intelligence have improved risk stratification and influenced contemporary international guidelines.

**Objective:**

To synthesize the most recent evidence on the diagnosis, risk stratification, and management of IPMNs, with emphasis on comparing the Fukuoka (2017), European (2018), and Kyoto (2024) guidelines, and on the integration of emerging technologies.

**Methods:**

A structured narrative review was conducted based on major international clinical guidelines and systematic reviews identified through PubMed/MEDLINE and Embase. Eligible publications in English from 2020 to 2025 addressing clinical, radiologic, molecular, and management-related aspects of IPMNs were included.

**Results:**

Diagnosis primarily relies on magnetic resonance imaging and endoscopic ultrasound, supplemented by fine-needle aspiration, molecular analysis of cyst fluid, and emerging tools such as radiomics and artificial intelligence models. International guidelines differ in risk criteria, anatomic thresholds, and recommended surveillance intervals, directly influencing decisions between follow-up and surgical resection. Minimally invasive techniques, including EUS-guided radiofrequency ablation and intralesional chemoablation, are under investigation as alternative options for patients who are not surgical candidates.

**Conclusion:**

The management of IPMNs should be individualized and multidimensional, integrating clinical, radiologic, and molecular information within the framework of updated guidelines. Active surveillance is safe for low-risk lesions, whereas surgery remains indicated for patients with high-risk stigmata. Emerging technologies including radiomics, artificial intelligence, and next-generation sequencing, are expected to enhance diagnostic precision and support personalized therapeutic decision-making.

## HIGHLIGHTS

•International guidelines (Fukuoka, European, Kyoto) differ in risk definitions and surveillance thresholds.

•Advanced tools, such as radiomics, molecular markers, and artificial intelligence, enhance diagnostic accuracy.

•Integrating clinical, radiologic, and molecular data improves individualized management.

•Active surveillance is safe for low-risk lesions; surgery is indicated for patients with high-risk stigmata.

•Minimally invasive EUS-guided therapies represent emerging alternatives for selected patients.

## Abstract (pt)

RESUMO

**Contexto:**

As neoplasias papilares mucinosas intraductais do pâncreas (IPMNs) constituem a principal causa de cistos pancreáticos detectados por imagem, apresentando amplo espectro biológico e risco variável de progressão maligna. Avanços em métodos de imagem, ultrassonografia endoscópica, biópsia, análise molecular e inteligência artificial têm aperfeiçoado a estratificação de risco e influenciado as diretrizes internacionais. **Objetivo:** Sintetizar as evidências mais recentes sobre diagnóstico, estratificação de risco e manejo das IPMNs, comparando as recomendações das diretrizes de Fukuoka (2017), Europeias (2018) e Kyoto (2024), com ênfase na incorporação de novas tecnologias.

**Métodos:**

Conduziu-se uma revisão narrativa estruturada da literatura, fundamentada em diretrizes internacionais e revisões sistemáticas identificadas nas bases PubMed/MEDLINE e Embase, publicadas em inglês entre 2020 e 2025. Foram incluídos estudos clínicos, radiológicos e moleculares relevantes para diagnóstico, estratificação de risco e manejo das IPMNs.

**Resultados:**

O diagnóstico baseia-se principalmente em ressonância magnética e ultrassonografia endoscópica, complementadas por punção aspirativa, análise molecular do fluido cístico e ferramentas emergentes como radiômica e modelos de inteligência artificial. As diretrizes internacionais apresentam diferenças quanto aos critérios de risco, limiares anatômicos e intervalos de vigilância, influenciando a decisão entre seguimento e pancreatectomia. Técnicas minimamente invasivas, como ablação por radiofrequência guiada por EUS e quimioablação intralesional, estão em avaliação e podem representar alternativas para pacientes não candidatos à cirurgia.

**Conclusão:**

O manejo das IPMNs deve ser individualizado, integrando informações clínicas, radiológicas e moleculares conforme as diretrizes atualizadas. A vigilância é segura para lesões de baixo risco, enquanto a cirurgia permanece indicada para casos com estigmas de alto risco. Tecnologias emergentes incluindo radiômica, inteligência artificial e sequenciamento de nova geração tendem a aprimorar a tomada de decisão e favorecer abordagens mais personalizadas.

## INTRODUCTION

Pancreatic cysts have been diagnosed with increasing frequency, largely due to the widespread use of high-resolution imaging modalities, particularly magnetic resonance imaging and magnetic resonance cholangiopancreatography (MRCP). It is estimated that the prevalence of incidental pancreatic cysts rises progressively with age, reaching values close to 40% in individuals over 60 years old, as demonstrated in large-scale population studies^1^ ^,^ ^2^. The accurate identification and appropriate management of these lesions are essential to reduce the morbidity and mortality associated with pancreatic adenocarcinoma.

Within this spectrum of lesions, intraductal papillary mucinous neoplasms (IPMNs) stand out due to their malignant transformation potential. Similar to mucinous cystic neoplasms^3^, IPMNs represent precursor lesions of pancreatic adenocarcinoma, one of the malignancies with the poorest prognosis worldwide^1^ ^,^ ^4^ ^,^ ^5^. IPMNs are characterized by ductal dilatation associated with mucin production and may involve the main duct, the branch ducts, or both, resulting in distinct risk profiles. The incidental detection of these lesions poses a significant clinical challenge: determining which cases require structured surveillance and which necessitate surgical intervention, while maintaining a balance between preventing invasive carcinoma and minimizing unnecessary surgeries.

Over the past decades, the diagnosis of IPMNs has been enhanced by substantial advances in imaging, endoscopy, and molecular biology. Recent studies highlight the role of techniques such as EUS-FNB, through-the-needle biopsy^6^, molecular analysis of cyst fluid, radiomics, and artificial intelligence^7^ ^-^ ^12^. These tools have contributed to more accurate risk stratification and increasingly individualized management strategies.

Multiple international guidelines provide direction for the management of IPMNs, each employing its own set of criteria: the 2017 Fukuoka Guidelines^4^, the 2018 European Guidelines^5^, and, more recently, the 2024 Kyoto Guidelines^13^. Despite notable progress, these recommendations differ in anatomical criteria, risk thresholds, and surveillance intervals, influencing risk interpretation and surgical decision-making^1^ ^,^ ^14^.

This review integrates the principal evidence published between 2020 and 2025, complemented by established international guidelines, with emphasis on diagnostic advances, risk stratification, surgical indications, and surveillance strategies for IPMNs. Despite recent progress, there remains a clear need for updated syntheses that incorporate guidelines and emerging technologies, particularly in view of the rapid evolution of diagnostic tools and the heterogeneity among international recommendations.

## METHODS

### Study type and methodological rationale

This is a structured narrative review, conducted in accordance with the principles of the SANRA (Scale for the Assessment of Narrative Review Articles) scale^15^, without a preregistered protocol and without application of the full PRISMA (Preferred Reporting Items for Systematic Reviews and Meta-Analyses) checklist, since no exhaustive search or formal quantitative synthesis was performed. The PRISMA flowchart employed is simplified and adapted, serving solely to ensure transparency of the screening process. The focus was a critical and comparative synthesis of guidelines and recent evidence regarding risk stratification and management of IPMNs.

### Information sources and databases

The literature search was conducted in PubMed/MEDLINE and Embase databases.

These sources were selected due to their broad coverage of gastroenterology, pancreatic surgery, endoscopy, and diagnostic imaging.

Sources of grey literature, preprints, conference abstracts, or materials lacking peer review were not included.

### Search strategy

The literature search was conducted in the PubMed/MEDLINE and Embase databases, using MeSH/Emtree descriptors and Boolean operators, covering the period from January 1, 2020, to July 30, 2025. Two reviewers independently screened titles, abstracts, and full texts, applying predefined inclusion criteria (international guidelines, systematic reviews, and original studies relevant to the diagnosis or management of IPMNs).

The search syntax applied in each database is presented in Table 1.

**TABLE 1:** Search strategy (PubMed/MEDLINE and Embase).

Database	Search strategy
PubMed/MEDLINE	(“Intraductal Papillary Mucinous Neoplasm” OR “IPMN”) AND (“diagnosis” OR “management” OR “malignant transformation” OR “surveillance”)
Embase	(“Intraductal Papillary Mucinous Tumor” OR “IPMN”) AND (“imaging” OR “follow-up” OR “treatment” OR “risk stratification”)

### Study selection process

Screening was performed by two independent reviewers (CFT and HCT) in three stages: title review, abstract review, and full-text assessment.

Duplicate records were identified and automatically removed using Zotero software, ensuring standardization and reproducibility in the selection process.

**FIGURE 1:**
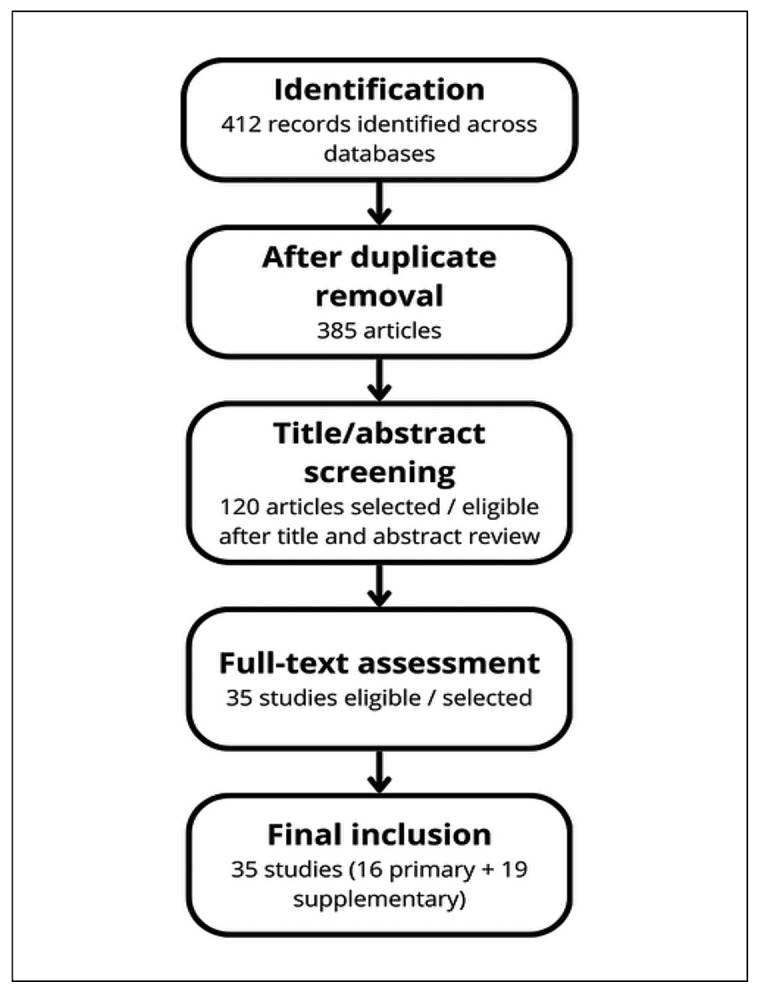
Simplified PRISMA flow diagram.

Note: this PRISMA flow diagram represents a simplified adaptation appropriate for structured narrative reviews. It does not correspond to the full protocol of a systematic review or meta-analysis, as no exhaustive searches, standardized data extraction, formal risk-of-bias assessment, or quantitative synthesis were performed.

Figure 1 summarizes the findings in a simplified PRISMA flowchart.

### Inclusion criteria

•Studies published between January 1, 2020, and July 30, 2025, identified through the structured search;
•International guidelines and seminal studies published prior to this period, included complementarily for conceptual contextualization and comparison across recommendations;
•Publications in English - this restriction was adopted because the main international guidelines (Fukuoka 2017, European 2018, and Kyoto 2024) and most high-impact studies on IPMNs are produced in this language. National guidelines in Portuguese generally reproduce these recommendations without providing novel primary data; therefore, limiting inclusion to English did not compromise the analysis;
•Studies involving patients with IPMNs confirmed by imaging, histology, or surgery;
•Publications related to diagnosis, risk stratification, clinical management, surveillance, or surgical indication of IPMNs;
•Systematic reviews, original studies, cohort studies, meta-analyses, and clinical guidelines.

### Exclusion criteria

Studies focused on other pancreatic diseases;

Single case reports;

Animal model studies;

Publications lacking clinically relevant outcomes;

Redundant or repetitive narrative reviews;

Publications without access to the complete manuscript.

The methodological quality of the studies was evaluated descriptively, in accordance with the format of a structured narrative review. Consolidated tools such as AMSTAR-2^16^ for systematic reviews, QUADAS-2 (Quality Assessment of Diagnostic Accuracy Studies-2)^17^ for diagnostic accuracy studies, and SANRA^15^ to guide overall review consistency were used only in an adapted, interpretative manner. No formal scoring, grading, or exclusion of articles was performed based on these instruments. The aim was to support the critical appraisal of the evidence and to contextualize methodological limitations, particularly in the discussion section.

### Data synthesis

Data synthesis was performed descriptively and narratively, integrating clinical, radiological, and molecular findings reported by the included publications. No standardized data extraction, combined statistical analysis, or meta-analysis was undertaken, as this was not a systematic review. Studies were grouped by theme (diagnosis, risk stratification, surgical management, surveillance, and emerging technologies).

## RESULTS

### Imaging Techniques in the Diagnosis of IPMN

The diagnosis of intraductal papillary mucinous neoplasms (IPMNs) relies primarily on imaging modalities, which enable assessment of morphology, ductal communication, presence of mural nodules, and associated risk features. Magnetic resonance imaging with cholangiopancreatography (MRI/MRCP) is recommended as the initial examination by international guidelines due to its excellent delineation of the pancreatic ducts and its higher sensitivity for subtle alterations suggestive of advanced dysplasia^1^ ^,^ ^4^ ^,^ ^5^ ^,^ ^13^.

Contrast-enhanced computed tomography (CT) plays a complementary role, particularly in the evaluation of calcifications, solid components, and extra pancreatic disease. Although less sensitive than MRI for detecting small mural nodules, CT maintains high accuracy for staging and investigation of vascular invasion^2^ ^,^ ^14^.

Endoscopic ultrasonography (EUS), with or without fine-needle aspiration (FNA), is considered a central tool in the evaluation of IPMNs. The method provides superior spatial resolution for identifying mural nodules, cyst wall thickening, and vascular patterns suggestive of malignant transformation, in addition to enabling analysis of cyst fluid (CEA, cytology) when indicated^1^ ^-^ ^3^ ^,^ ^5^ ^,^ ^14^.

In recent years, advanced techniques have expanded the diagnostic capabilities of EUS. Contrast-enhanced EUS (EUS-CE) improves discrimination between debris and true nodules, increasing accuracy for detecting vascularized lesions associated with a higher risk of malignancy. Confocal laser endomicroscopy^6^ and through-the-needle microforceps biopsy^6^ offer improved preoperative histological characterization, particularly in diagnostically challenging cases^2^ ^,^ ^3^ ^,^ ^8^ ^,^ ^9^ ^,^ ^18^.

Radiomics and artificial intelligence (AI) have emerged as promising approaches for quantitative image analysis. Recent studies have shown that radiomics-based models extract subtle patterns not perceptible to the human eye, with performance superior to conventional methods for risk stratification. AI tools have demonstrated potential for malignancy prediction, morphological classification, and multimodal data integration (imaging, clinical variables, and molecular biology)^19^ ^-^ ^22^.

Taken together, the integrated combination of MRI/MRCP, advanced EUS, cyst fluid biomarkers, and computational techniques represents the most accurate diagnostic approach and aligns with the current standard of care for IPMNs.

### Molecular biomarkers and cyst fluid analysis

Cyst fluid analysis obtained through EUS-FNA complements the morphological assessment of IPMNs by assisting in differentiating mucinous from non-mucinous lesions and refining risk stratification. Among the available markers, intracystic CEA (Carcinoembryonic Antigen) remains widely used, although its isolated diagnostic value is limited. Elevated levels suggest mucinous etiology, but do not predict malignancy or indicate surgery, which is why current guidelines recommend interpreting CEA results in conjunction with clinical and radiological findings^10^ ^,^ ^11^ ^,^ ^23^.

Intracystic glucose <50 mg/dL has demonstrated superior performance compared with CEA (Carcinoembryonic Antigen) for distinguishing mucinous from non-mucinous cysts, with higher accuracy, lower interlaboratory variability, and simpler and more cost-effective execution. Recent studies reinforce its growing utility as the preferred biochemical test in many centers^12^ ^,^ ^24^.

Serum biomarkers such as CA 19-9 (Cancer Antigen 19-9) maintain a complementary role. Significant elevations, particularly when associated with jaundice, mural nodules, or main duct dilatation, may indicate a higher risk of advanced dysplasia and justify intensified evaluation or surveillance^5^ ^,^ ^13^ ^,^ ^25^.

Molecular analysis of cyst fluid by next-generation sequencing (NGS) has become one of the most informative tools in preoperative evaluation. KRAS and GNAS mutations are strongly associated with mucinous pathology and show high sensitivity for identifying IPMNs^10^ ^,^ ^11^ ^,^ ^23^. Alterations in TP53, SMAD4, RNF43, and PIK3CA are linked to high-grade dysplasia or invasive carcinoma, providing relevant prognostic information^6^ ^,^ ^10^ ^,^ ^11^ ^,^ ^23^. The combination of early mutations (KRAS/GNAS) with progression-associated markers (TP53/PIK3CA/SMAD4/RNF43) offers excellent performance for identifying high-risk lesions and is increasingly incorporated into modern recommendations, such as the 2024 Kyoto Guidelines^6^ ^,^ ^10^ ^,^ ^11^ ^,^ ^13^ ^,^ ^23^.

Overall, no single biomarker is sufficient to determine management. Integrating biochemical, cytological, and molecular findings with clinical and imaging criteria remains the most accurate approach for risk stratification and decision-making in the management of IPMNs.

The performance of the principal diagnostic modalities is summarized in Table 2, including sensitivity, specificity, and accuracy extracted from studies published between 2020 and 2025. Results are presented as ranges, reflecting heterogeneity among centers, methodological designs, and diagnostic criteria.

**TABLE 2:** Diagnostic performance of the main techniques used in the evaluation of IPMN.

Technique	Sensitivity (%)	Specificity (%)	Accuracy (%)
EUS-FNA + cytology^10^ ^,^ ^12^ ^,^ ^23^	50-75	80-95	70-85
EUS-guided microforceps biopsy^10^ ^,^ ^11^	70-82	83-90	88-95
Radiomics / Artificial intelligence^7^ ^,^ ^20^	82-92	80-90	85-92
Cyst fluid glucose^12^ ^,^ ^24^ ^,^ ^25^	85-95	80-95	85-92
Cyst fluid CEA^11^ ^,^ ^12^ ^,^ ^23^ ^,^ ^24^	63-80	78-95	70-82
Cyst fluid NGS^6^ ^,^ ^10^ ^,^ ^11^ ^,^ ^23^	90-97	92-98	94-98

EUA-FNA - Endoscopic ultrasonography (EUS), with fine-needle aspiration (FNA). Note: Values represent aggregated ranges from multiple studies and may vary across centers.

### Clinical criteria

Clinical findings are a fundamental component of risk stratification in IPMNs and complement the radiological criteria defined by the Fukuoka, European, and Kyoto guidelines^4^ ^,^ ^5^ ^,^ ^13^. Although many lesions are asymptomatic, certain clinical features increase the likelihood of high-grade dysplasia or invasive carcinoma.

Recurrent epigastric pain occurs in a variable proportion of patients with IPMNs and may reflect ductal distension, inflammation, or partial obstruction. It is considered a worrisome feature, particularly when associated with ductal abnormalities on EUS/MRI^1^ ^,^ ^4^ ^,^ ^5^ ^,^ ^14^.

Jaundice, generally resulting from biliary compression by lesions located in the pancreatic head, is one of the strongest indicators of malignancy and is associated with high rates of invasive carcinoma in main-duct IPMNs. For this reason, it remains a high-risk stigma warranting surgical intervention in operable patients^4^ ^,^ ^5^ ^,^ ^13^.

Pancreatitis may arise from obstruction of the pancreatic duct by mucin and is recognized as a warning sign, particularly in branch-duct IPMNs. In cases of recurrent pancreatitis, detailed EUS evaluation in specialized centers is recommended^1^ ^,^ ^4^ ^,^ ^5^ ^,^ ^14^.

Weight loss, although nonspecific, may suggest neoplastic progression or associated pancreatic insufficiency. European guidelines emphasize that systemic symptoms should prompt intensified surveillance^1^ ^,^ ^5^ ^,^ ^14^.

Patients with a family history of pancreatic neoplasia or germline syndromes carry an intrinsically higher risk of malignant transformation and require differentiated surveillance. Recent studies reinforce the increased risk of progression in these groups^6^ ^,^ ^13^ ^,^ ^25^.

### Risk stratification

Risk stratification in IPMN aims to identify lesions with a greater likelihood of progressing to high-grade dysplasia or invasive carcinoma, thereby guiding the choice between surveillance and surgical treatment. Recent evidence (2020-2025) demonstrates that the risk of malignant progression is heterogeneous and depends on the integration of anatomical, clinical, and molecular factors, including imaging findings, symptoms, CA 19-9 levels, and the genetic profile of cyst fluid^1^ ^,^ ^7^ ^,^ ^25^ ^-^ ^29^.

Contemporary studies show that low-risk IPMNs exhibit a cumulative malignancy probability of approximately 2-3% at 5 years, whereas lesions involving the main duct or harboring a true mural nodule may reach up to 15% risk over 10-15 years^7^ ^,^ ^26^ ^-^ ^29^. These data reinforce the need for structured, guideline-based follow-up.

International approaches converge on a common framework:

- Fukuoka 2017 prioritizes anatomical criteria^13^;
- Europe 2018 emphasizes multidisciplinary decision-making and complementary use of cytology and biomarkers;
- Kyoto 2024 integrates molecular prediction and AI into traditional clinical models, providing more personalized stratification^4, 5, 13, 28, 30, 31^.

International risk stratification is primarily grounded in three widely adopted guidelines, reviewed below.

### Comparison of international guidelines(Fukuoka 2017, Europe 2018, Kyoto 2024)

International guidelines constitute the primary foundation for the modern management of IPMNs, guiding surveillance, risk stratification, and surgical indication. Although they share common principles, they differ in the depth of evaluation, the role of biomarkers, and the incorporation of emerging technologies.

### Fukuoka guidelines (2017)^4^

Fukuoka remains the most widely used guideline due to its simplicity, robustness, and broad international validation. It is based essentially on anatomical and radiological criteria^4^.

High-risk stigmata (HRS) - strong surgical indication:

- Obstructive jaundice associated with an IPMN of the pancreatic head;
- Enhancing mural nodule ≥5 mm;
- Main pancreatic duct (MPD) dilation >10 mm.

Worrisome Features^13^ - Require complementary EUS evaluation:

- Cyst ≥3 cm;
- Thickened or irregularly enhancing cyst wall;
- MPD between 5-9 mm;
- Associated pancreatitis;
- Suspicious lymph nodes;
- Rapid cyst growth (≥5 mm/6 months or ≥10 mm/year).

**In summary:** Fukuoka is objective and widely applicable, serving as the basis for surveillance worldwide^4^.

### European guidelines (2018)^5^

The European guidelines maintain the fundamental principles of Fukuoka but adopt a more integrated and multidisciplinary approach. They emphasize expanded use of complementary methods and greater flexibility in surveillance, incorporating additional elements of risk stratification^5^.

Main differences compared with Fukuoka include:

- Mandatory multidisciplinary discussion involving radiology, endoscopy, surgery, and pathology;
- Greater use of EUS ± FNA, particularly for characterization of mural nodules and acquisition of cyst fluid;
- Enhanced valuation of cytology and biomarkers, including CEA and KRAS/GNAS mutations;
- Recognition of the potential of radiomics, with increasing attention to complex patterns identified on MRI^1^ ^,^ ^5^ ^,^ ^19^ ^,^ ^20^.

**Dynamic surveillance:** surveillance intervals are modulated according to cyst size and individual risk, allowing personalized adjustment of clinical management^5^ ^,^ ^28^ ^-^ ^30^.

**In summary:** the European guidelines are more flexible and adaptable to different clinical contexts, reinforcing the role of complementary testing and advanced technologies when available^5^.

### Kyoto guidelines (2024)^13^

The Kyoto guidelines represent the most modern and comprehensive evolution of existing recommendations, incorporating dynamic clinical findings, molecular biomarkers, and computational technologies.

Main updates:

### 1. Clinical and laboratory factors:

- Elevated CA 19-9 as an independent risk marker;
- New-onset diabetes mellitus;
- Family history of pancreatic cancer;
- Explicit consideration of age, comorbidities, and life expectancy.

### 2. Biomarkers and genetics:

- Inclusion of KRAS and GNAS mutations (mucinous characterization);
- Emphasis on TP53, SMAD4, RNF43, and PIK3CA mutations, which are associated with advanced dysplasia^6^ ^,^ ^10^ ^,^ ^11^ ^,^ ^13^ ^,^ ^23^.

### 3. Integrated emerging technologies:

- Radiomics applied to MRI/EUS;
- Artificial intelligence-based models for risk prediction;
- Personalized surveillance algorithms^6^ ^,^ ^10^ ^,^ ^11^ ^,^ ^13^ ^,^ ^23^.

**In summary:** Kyoto is the most comprehensive guideline and the one most aligned with personalized medicine, ideal for centers with a high level of technological resources and diagnostic capability^13^.

### Comparative synthesis

The three guidelines converge on essential points: structured surveillance, the central role of MRI/EUS, and surgical indication based on anatomical criteria^4^ ^,^ ^5^ ^,^ ^13^ ^,^ ^21^.

They differ, however, in depth:

- Fukuoka 2017: objective and primarily morphology based.
- Europe 2018: broader, integrating cytology, biomarkers, and a multidisciplinary approach.
- Kyoto 2024: incorporates genetics, AI, and dynamic clinical evaluation, reflecting the most advanced level of personalized medicine.

Table 3 provides a structured comparison of the Fukuoka 2017, Europe 2018, and Kyoto 2024 guidelines, highlighting convergences and divergences in anatomical criteria, clinical risk factors, definitions of high-risk stigmata and worrisome features, use of biomarkers, and surgical recommendations.

**TABLE 3:** Comparison of major international guidelines for IPMN management.

Aspect Evaluated	Fukuoka 2017^4^ - Anatomical focus	European 2018^5^ - Multidisciplinary focus	Kyoto 2024^13^ - Personalized and molecular focus
Conceptual basis	Morphological criteria. (HRS and WF).	Maintains Fukuoka framework, adding cytology and biomarkers.	Integrates clinical, genetic, radiomic, and AI data.
High-Risk Stigmata (HRS)	Jaundice, mural nodule ≥5 mm, MPD >10 mm è surgery.	Same HRS; reinforces high-grade cytology as a strong indicator.	Maintains HRS and adds factors such as elevated CA 19-9 and clinical risk.
Worrisome Features (WF)	Cyst ≥3 cm, thickened wall, MPD 5-9 mm, pancreatitis, suspicious lymph nodes.	Adopts WF; uses EUS ± FNA, cytology, and biomarkers to refine risk.	Maintains WF but adds biomarkers (KRAS/GNAS/TP53/SMAD4) and dynamic clinical data (new-onset diabetes, CA 19-9).
Surveillance (branch-duct IPMN)	MRI/EUS every 6-24 months depending on size.	More flexible and individualized intervals.	Surveillance adjusted using predictive algorithms (AI) and individualized risk.
Surgical indication	≥1 HRS; consider in multiple WF or disease progression.	Maintains criteria but requires multidisciplinary decision-making.	Integrates surgical + molecular risk; greater caution in elderly or comorbid patients.
Use of biomarkers	Cyst fluid CEA and serum CA 19-9 as auxiliary tests.	Expands use of KRAS/GNAS; consider NGS when available.	Includes full panels (KRAS, GNAS, TP53, SMAD4, RNF43); incorporates radiomics and AI.
General approach	Simple and structured system; radiology centered.	Broader approach integrating cytology and molecular testing.	Most modern guidelines: personalization, multimodality, and risk prediction.

AI: artificial intelligence. CA 19-9: Cancer Antigen 19-9. CEA: Carcinoembryonic Antigen. EUS: endoscopic ultrasonography. FNA: fine-needle aspiration. IPMN: Intraductal papillary mucinous neoplasm. MPD: main pancreatic duct. MRI: magnetic resonan­ce Imaging. NGS: next-generation sequencing.

### Therapeutic management: surveillance, surgery, and minimally invasive alternatives

The management of IPMNs is based on estimating the individual risk of malignant progression and integrates clinical, radiological, and, when available, molecular findings. The international guidelines, Fukuoka 2017^4^, Europe 2018^5^, and Kyoto 2024^13^, provide structured criteria that guide the choice between active surveillance, additional investigation with EUS, or surgical indication.

### Active surveillance

Active surveillance is recommended for patients without High-Risk Stigmata and without Worrisome Features, with the aim of monitoring morphological changes suggestive of advanced dysplasia. Surveillance intervals are primarily based on cyst size according to international consensus^4^ ^,^ ^5^ ^,^ ^13^ ^,^ ^28^ ^-^ ^30^:

- <1 cm: MRI/CT every 2-3 years;
- 1-2 cm: annual MRI;
- 2-3 cm: MRI or EUS every 6-12 months;
- >3 cm: close surveillance, preferably with serial EUS.

Dynamic clinical markers, such as rising CA 19-9, new-onset diabetes, accelerated cyst growth, and episodes of associated pancreatitis, may indicate higher risk of progression and prompt earlier intervention^6^ ^,^ ^13^ ^,^ ^25^ ^,^ ^28^ ^,^ ^29^.

Contemporary studies show that, when patients are appropriately stratified, surveillance has a low progression rate and is safe for branch-duct IPMNs without high-risk criteria^7^ ^,^ ^26^ ^-^ ^29^.

### Surgical indication

Surgery is indicated when there is strong suspicion of malignancy, particularly in the presence of High-Risk Stigmata according to Fukuoka 2017^4, 5, 13^:

- Obstructive jaundice associated with the cyst;
- Mural nodule ≥5 mm;
- Main pancreatic duct dilation >10 mm.

Patients with Worrisome Features require EUS to assess mural nodules, cyst wall thickening, and abnormal vascularity. Surgical indication depends on confirmation of suspicious findings by EUS^2^ ^,^ ^4^ ^,^ ^5^ ^,^ ^9^ ^,^ ^13^.

The choice of surgical technique varies according to lesion location:

- **Pancreaticoduodenectomy** - for lesions in the pancreatic head;
- **Distal pancreatectomy** - for lesions in the body or tail;
- **Total pancreatectomy** - for multifocal disease or diffuse dilation of the main duct.

Despite its potentially curative nature, pancreatic surgery carries considerable morbidity. Therefore, recent guidelines emphasize individualized decision-making, considering age, comorbidities, and patient preference^1^ ^,^ ^5^ ^,^ ^32^ ^-^ ^35^.

### Minimally invasive alternatives (inoperable patients)

In patients with surgical contraindications or those who decline operative intervention, minimally invasive techniques guided by endoscopic ultrasonography (EUS) have been evaluated as experimental options. Among these, intracystic chemoablation (e.g., with paclitaxel) and radiofrequency ablation (EUS-RFA) are the most prominent.

Recent observational studies suggest that these modalities may reduce the volume of selected cysts, particularly smaller and unilocular lesions. However, the number of enrolled patients remains small, selection criteria vary considerably among centers, and long-term oncologic follow-up is still limited^10^ ^,^ ^30^.

Reported complications include transient abdominal pain and mild pancreatitis, but the true rate of adverse events remains uncertain owing to the limited number of studies. International guidelines (Fukuoka 2017, Europe 2018, and Kyoto 2024) classify these techniques as non-standardized and recommend their use only within research protocols or in centers with specific expertise^4^ ^,^ ^5^ ^,^ ^13^.

Thus, at present, chemoablation and EUS-RFA do not replace structured surveillance or established surgical indications, and their use should be individualized in inoperable patients following multidisciplinary discussion.

### Multidisciplinary approach

The guidelines consistently emphasize that therapeutic decisions should be shared and guided by a multidisciplinary team (gastroenterology, radiology, surgery, and pathology), integrating imaging findings, dynamic clinical markers, molecular data when available, individual surgical risk, and patient preferences^5^ ^,^ ^13^ ^,^ ^31^ ^,^ ^35^.

This approach improves surveillance safety, avoids unnecessary pancreatectomies, and aligns management decisions with the patient’s true level of risk.

### Narrative synthesis of the 35 included studies (2020-2025)

The synthesis of the 35 studies published between 2020 and 2025 reveals consistent advances across three central axes in the management of IPMNs: imaging diagnostics, molecular characterization, and clinical outcomes related to surveillance or surgery.

### Advances in imaging methods

MRI/MRCP remains the initial examination of choice due to its excellent ductal delineation and superior ability to detect mural nodules and communication with the main pancreatic duct^1^ ^,^ ^2^ ^,^ ^7^ ^,^ ^19^ ^,^ ^20^.

EUS, particularly when combined with fine-needle aspiration (EUS-FNA), continues to be the main complementary modality for characterizing small mural nodules and cyst wall thickening. Advanced techniques such as contrast-enhanced EUS, elastography, micro forceps biopsy^6^, and confocal laser endomicroscopy have expanded diagnostic accuracy in differentiating low-grade from high-grade dysplasia^3^ ^,^ ^8^ ^,^ ^9^ ^,^ ^21^.

Radiomics and artificial intelligence models demonstrated performance superior to conventional methods in predicting advanced dysplasia and enabling individualized risk stratification^19^ ^,^ ^20^.

### Molecular studies and cyst fluid analysis

NGS-based analyses reaffirm the role of KRAS and GNAS as markers of mucinous differentiation, while mutations in TP53, SMAD4, RNF43, and PIK3CA are strongly associated with high-grade dysplasia and tumor progression^10^ ^,^ ^11^ ^,^ ^23^. The combination of early mutations with progression-associated mutations demonstrated superior accuracy in identifying higher-risk lesions^10^ ^,^ ^11^ ^,^ ^23^.

Among biochemical markers, intracystic CEA remains useful for distinguishing mucinous from non-mucinous cysts, although it does not independently predict malignancy or surgical indication^10^ ^,^ ^11^ ^,^ ^23^. Intracystic glucose demonstrated superior performance compared with CEA in multiple studies, being a simple, inexpensive, and extremely sensitive test^12^ ^,^ ^24^.

Serum CA 19-9 remains an important auxiliary marker, particularly when elevated in association with a dilated main duct or mural nodules^5^ ^,^ ^13^ ^,^ ^25^.

### Surveillance, surgery, and alternative therapies

Long-term surveillance series demonstrate a low rate of progression in appropriately stratified branch-duct IPMNs according to the Fukuoka, European, and Kyoto criteria^7^ ^,^ ^26^ ^-^ ^29^.

Surgical studies reinforce that, although pancreatectomy is effective for high-risk disease, postoperative morbidity remains significant, supporting selective surgical indication and rigorous multidisciplinary assessment^32^ ^,^ ^35^.

For inoperable patients, preliminary studies on endoscopic radiofrequency ablation (EUS-RFA) and chemoablation show promising outcomes in specialized centers, but with small sample sizes and lack of long-term oncologic follow-up, keeping these modalities as complementary and still experimental strategies^10^ ^,^ ^30^.

### Convergence of international guidelines

The combined analysis of the studies reinforces that the Fukuoka 2017, European 2018, and Kyoto 2024 guidelines converge in their anatomical criteria for high risk, but differ in the use of advanced biomarkers, radiomics, and artificial intelligence. Kyoto has emerged as the most modern guideline, integrating molecular tools and computational models^4^ ^,^ ^5^ ^,^ ^13^ ^,^ ^22^ ^,^ ^28^ ^,^ ^30^ ^,^ ^31^.

### Final summary

Taken together, the studies published between 2020 and 2025 support that the management of IPMNs should be individualized, combining imaging findings, biomarkers, and NGS when available, with a multidisciplinary approach and structured surveillance. The evidence points toward a clear movement toward personalized medicine, incorporating AI and predictive algorithms to optimize clinical decision-making^1, 4-6, 10, 11, 13, 14, 19, 20, 22, 23, 25, 28, 30, 31^.

## DISCUSSION

The management of intraductal papillary mucinous neoplasms (IPMNs) requires a multidisciplinary, personalized, and guideline-oriented approach, given the biological heterogeneity of these lesions, which may range from indolent alterations to invasive carcinoma. The major international guidelines, Fukuoka 2017, the 2018 European guidelines, and Kyoto 2024, provide a consolidated framework for risk stratification, guiding decision-making by integrating clinical, radiological, and, more recently, serum and molecular biomarkers^4^ ^-^ ^6^ ^,^ ^13^ ^,^ ^15^ ^-^ ^17^ ^,^ ^30^ ^,^ ^31^.

The findings of this review reinforce the central role of magnetic resonance cholangiopancreatography (MRI/MRCP) and endoscopic ultrasonography (EUS) in the structural evaluation of IPMNs. MRI/MRCP remains the preferred initial modality for defining ductal communication, disease extent, and the presence of mural nodules, whereas EUS, with or without fine-needle aspiration (FNA), is particularly useful for characterizing cyst-wall thickening, small nodules, and suspicious lymph nodes^1^ ^,^ ^2^ ^,^ ^4^ ^,^ ^5^ ^,^ ^13^ ^,^ ^14^ ^,^ ^20^ ^,^ ^21^.

Advanced techniques such as contrast-enhanced EUS, elastography, confocal endomicroscopy, and through-the-needle microforceps biopsy^6^ have demonstrated additional improvements in diagnostic accuracy in recent series, particularly for indeterminate cysts^2^ ^,^ ^3^ ^,^ ^8^ ^,^ ^11^ ^,^ ^18^ ^,^ ^23^.

The incorporation of radiomics and artificial intelligence (AI) represents one of the major diagnostic advances of the past decade. Models based on CT, MRI, or EUS have demonstrated superior ability to identify texture and enhancement patterns associated with advanced dysplasia compared with visual assessment alone^7^ ^-^ ^9^ ^,^ ^20^ ^,^ ^21^. Radiomics tools powered by machine learning have shown particularly promising performance, achieving higher predictive accuracy for malignancy or progression, especially in strategies that integrate multiple imaging modalities^7^ ^,^ ^9^ ^,^ ^20^. These findings indicate that, in the near future, computational tools may significantly support decisions related to surveillance or surgical indication, particularly in specialized centers with greater technological resources.

Despite these advances, the clinical implementation of radiomics and artificial intelligence models still faces important limitations. Available studies exhibit heterogeneity in imaging acquisition protocols, segmentation methods, and algorithms employed, which increases the risk of overfitting and compromises reproducibility across centers. Moreover, external multicenter validation remains limited, and the adoption of these technologies depends on adequate computational infrastructure and specialized teams, often available only in high-resource referral centers. Thus, although promising, these tools should be interpreted with caution and, in the current landscape, remain predominantly restricted to research environments.

In the field of biomarkers, the findings of this review confirm that intracystic CEA remains useful for distinguishing mucinous from non-mucinous cysts, but is insufficient to predict malignancy or guide surgical decision-making on its own, in accordance with the European and Kyoto guidelines^5^ ^,^ ^10^ ^,^ ^11^ ^,^ ^13^ ^,^ ^23^. Intracystic glucose has emerged as a simple, low-cost alternative with consistently superior performance for mucinous cyst characterization, whereas serum markers such as CA 19-9 remain relevant as warning indicators when elevated, particularly in association with jaundice, a mural nodule, or ductal dilation^25^ ^,^ ^30^ ^,^ ^32^.

Molecular analysis through next-generation sequencing (NGS) constitutes the most advanced component of risk stratification. KRAS and GNAS mutations are strongly associated with mucinous differentiation, whereas alterations in TP53, SMAD4, RNF43, and PIK3CA are related to high-grade dysplasia or invasive carcinoma^12^ ^,^ ^24^. Multicenter studies have shown that panels combining initiation mutations (KRAS/GNAS) with progression mutations (TP53/SMAD4/PIK3CA/RNF43) significantly enhance preoperative accuracy, and this approach is already reflected in the latest Kyoto criteria^10^ ^-^ ^13^ ^,^ ^24^.

Regarding natural history, long-term cohort studies show that side-branch IPMNs, when adequately stratified, present a relatively low cumulative risk of malignant transformation, with progression rates below 2-3% at 5 years and approximately 10-15% over longer follow-up periods, particularly in the presence of a dilated main pancreatic duct or a true mural nodule^2^ ^,^ ^22^ ^,^ ^25^ ^,^ ^28^ ^,^ ^34^. These findings support active surveillance in low-risk patients, especially in older adults or individuals with multiple comorbidities^16^ ^,^ ^25^ ^,^ ^28^ ^,^ ^31^ ^,^ ^35^.

Pancreatectomy remains the standard treatment for IPMNs with high-risk stigmata or confirmed worrisome features, given the strong association of these criteria with high-grade dysplasia or invasive carcinoma^4^ ^,^ ^5^ ^,^ ^13^ ^,^ ^17^ ^,^ ^30^ ^,^ ^33^. However, morbidity associated with the procedure remains significant, including risks of postoperative pancreatic fistula, gastroparesis, and pancreatogenic diabetes, requiring careful assessment of the risk-benefit balance, particularly in elderly or frail patients^1^ ^,^ ^17^ ^,^ ^22^ ^,^ ^30^ ^,^ ^33^. In this context, recent guidelines reinforce the importance of multidisciplinary decision-making and individualized risk stratification, aligning therapeutic choices with both safety and effectiveness^6^ ^,^ ^16^ ^,^ ^17^ ^,^ ^22^ ^,^ ^31^ ^,^ ^33^.

In resource-limited settings, full implementation of international recommendations must be adapted to local availability of imaging and expertise. In such contexts, the management of IPMNs may rely primarily on standardized MRI, conventional EUS, serial assessment of CA 19-9, and established morphological criteria from existing guidelines. Advanced techniques such as EUS-TTNB (EUS-guided Through-the-Needle Biopsy), nCLE (needle-based Confocal Laser Endomicroscopy), radiomics, NGS, and multimodal testing should be reserved for selected cases or referred to specialized centers, ensuring that decision-making reflects the actual capabilities of the service and prioritizes patient safety^5^ ^,^ ^6^ ^,^ ^13^.

For patients who are inoperable or decline surgery, minimally invasive EUS-guided techniques, such as intracystic chemoablation and endoscopic radiofrequency ablation (EUS-RFA), have been investigated in observational series, demonstrating meaningful volumetric responses and an acceptable safety profile in experienced centers^8^ ^-^ ^11^ ^,^ ^23^ ^,^ ^28^. Nevertheless, given the limited number of multicenter studies, the absence of randomized trials, and insufficient long-term oncologic follow-up, these approaches should be regarded as complementary strategies for selected cases and do not constitute therapeutic alternatives equivalent to surgical resection in current guidelines^16^ ^,^ ^17^ ^,^ ^28^ ^,^ ^30^.

Despite the growing interest in these modalities, current evidence remains limited. Available studies involve small cohorts, lack randomized trials, exhibit methodological heterogeneity, and require longer follow-up to adequately assess safety and efficacy. Complications such as pancreatitis, persistent pain, and ductal strictures have been reported, reinforcing that these techniques do not replace established surgical indications nor guideline-based structured surveillance. Consequently, their use should be restricted to experienced centers and to patients who are not candidates for surgery, and always within well-defined clinical protocols.

Overall, the synthesis of the 35 studies analyzed demonstrates increasing convergence among guidelines regarding the definition of high-risk criteria and the safety of active surveillance for low-risk lesions, while simultaneously highlighting the emerging role of molecular biomarkers, radiomics, and AI in constructing more refined risk models^4-9, 12, 13, 15-17, 20-22, 24, 25, 28^. The findings point to a progressive shift in the management of IPMNs toward a personalized medicine paradigm, in which therapeutic decisions are tailored to the clinical, morphological, and molecular profile of each patient.

In addition, to minimize selection bias during the search and screening phases, the Zotero software was used for systematic identification and removal of duplicate records, ensuring methodological accuracy and consistency in the selection of included studies.

### Limitations

This review presents limitations inherent to its narrative design, including the absence of a formal meta-analysis, methodological heterogeneity among the included studies, and the inability to perform a combined quantitative synthesis^3^ ^,^ ^26^. A formal risk-of-bias assessment (QUADAS-2, AMSTAR-2) was not fully applied, serving only as interpretive guidance rather than as a basis for exclusion.

Linguistic bias is also possible due to the inclusion of English-language publications only. This restriction was adopted because the major international reference guidelines and the most robust studies on IPMNs are predominantly published in English. To mitigate this limitation, well-established international guidelines, regardless of publication year, were incorporated, along with essential complementary literature for adequate contextualization.

A potential temporal bias is present as well, given that the structured search covered the period from 2020 to 2025. This was minimized by deliberately including earlier guidelines (Fukuoka 2017 and European 2018), which remain valid and widely applied in clinical practice.

**FIGURE 2:**
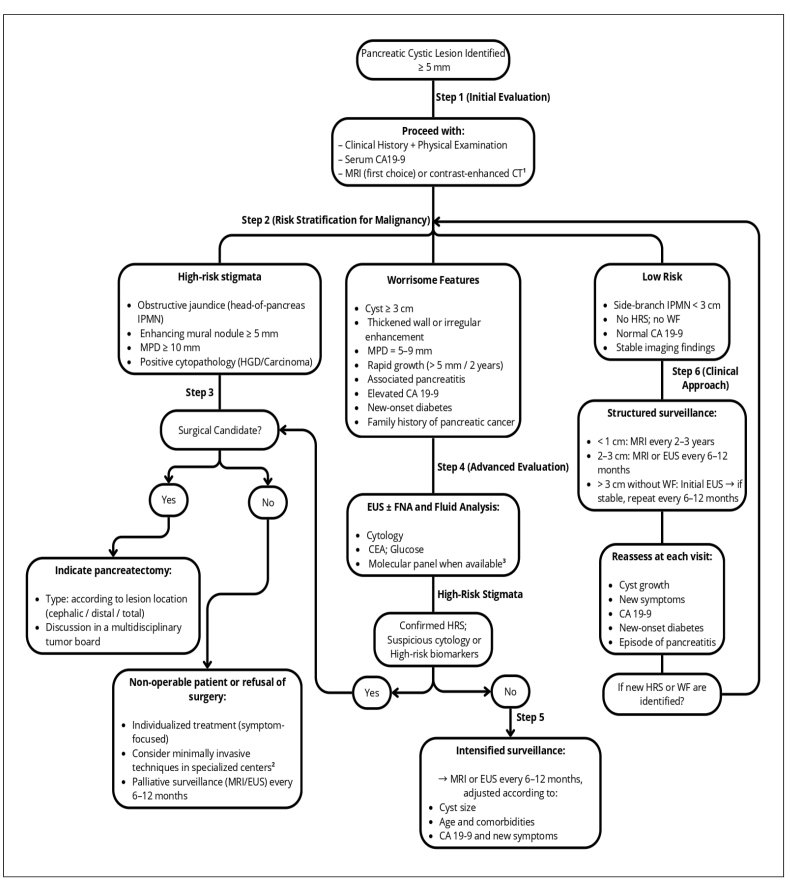
Integrated management flowchart for IPMNs based on Fukuoka 2017, European 2018, and Kyoto 2024 guidelines. The diagram synthesizes key decision steps, including initial clinical evaluation (1), risk stratification (2), indications for surgical management in high-risk cases (3), advanced assessment for intermediate-risk lesions (4), absence of HRS biomarkers and negative cytology (5), and size- and risk-based surveillance pathways (6).

Figure 2 presents an updated algorithm flowchart for the management of IPMNs, integrating anatomical criteria (HRS/WF), dynamic clinical factors (elevated CA 19-9, recent-onset diabetes), and molecular findings (KRAS/GNAS/TP53/SMAD4), with surveillance intervals derived from the Fukuoka, European, and Kyoto guidelines.

CA19-9: carbohydrate antigen 19-9. CEA: carcinoembryonic antigen. CT: computed tomography. EUS ± FNA: endoscopic ultrasound with fine-needle aspiration. HRS: high-risk stigmata. MPD: main pancreatic duct. IPMN: intraductal papillary mucinous neoplasm. MRI: magnetic resonance imaging. WS: worrisome features.

1 - Define: MPD-IPMN / Side-branch IPMN / Mixed type IPMN. 2 - EUS-RFA (endoscopic ultrasound-guided radiofrequency ablation) or intra lesional injection. 3 - KRAS, GNAS, TP53, SMAD4, RNF43, and PIK3CA.

Note: follow-up intervals were adapted from the Fukuoka 2017^4^, European 2018^5^, and Kyoto 2024^13^ guidelines. The additional dynamic factors incorporated into the algorithm, such as CA 19-9 elevation, recent-onset diabetes, and family history, were derived from evidence published between 2020 and 2025^6^ ^,^ ^22^ ^,^ ^25^ ^,^ ^28^ ^,^ ^29^ ^,^ ^31^ ^,^ ^32^ ^,^ ^35^, as detailed in the discussion section.

## CONCLUSION

The contemporary management of IPMNs relies on the balanced integration of clinical, radiological, serum biomarker, cyst fluid, and molecular findings, interpreted in accordance with major international guidelines. This combined approach enables more accurate distinction between low-risk lesions, suitable for safe surveillance, and those with a high potential for malignant progression, which benefit from selective surgical evaluation^4^ ^,^ ^5^ ^,^ ^10^ ^,^ ^13^ ^,^ ^16^ ^,^ ^17^ ^,^ ^22^ ^,^ ^25^ ^,^ ^28^ ^,^ ^30^.

Emerging technologies, including radiomics, machine learning models, and next-generation sequencing, show increasing potential to refine risk stratification, although their routine clinical integration still requires external validation, methodological standardization, and adequate infrastructure^7^ ^-^ ^9^ ^,^ ^12^ ^,^ ^20^ ^,^ ^21^ ^,^ ^24^. Clinical decisions should be individualized, considering surgical risk, comorbidities, dynamic clinical factors, and available diagnostic resources, in alignment with international recommendations.

Concurrently, minimally invasive EUS-guided therapies have emerged as promising alternatives for patients who are not surgical candidates; however, they still lack multicenter validation and robust oncologic follow-up before potential integration into current guidelines^8^ ^-^ ^11^ ^,^ ^23^ ^,^ ^28^.

Thus, surveillance and intervention strategies should be considered on a case-by-case basis, considering surgical risk, comorbidities, the evolution of clinical findings, and the local availability of diagnostic resources. In this context, the management of IPMNs is expected to evolve toward an increasingly personalized model, guided by the integration of technology, guidelines, and individualized risk assessment, while recognizing that such advances still require additional validation before universal implementation.

## Data Availability

Data availability statement: Not applicable. This study is a narrative review based exclusively on previously published literature and did not generate or analyze primary research data.
